# A review on molecular markers of *Plasmodium falciparum*

**DOI:** 10.1007/s12639-025-01840-0

**Published:** 2025-08-04

**Authors:** Wagaw Abebe, Dagmawi Woldesenbet

**Affiliations:** 1 Department of Medical Laboratory Sciences, College of Health Sciences, Woldia University, Woldia, Ethiopia; 2 Department of Medical Laboratory Sciences, College of Medicine and Health Sciences, Wachemo University, Hossana, Ethiopia

**Keywords:** Drug resistance, Marker, Review, *Plasmodium falciparum*

## Abstract

Malaria is an infectious disease caused by parasitic protozoans of the genus *Plasmodium.* Malaria control efforts on a global scale are in danger due to the emergence and spread of drug-resistant malaria. Despite stakeholders’ dedication to the prevention and treatment of malaria, the current state of global health does not offer an effective answer to the issue of drug resistance. Furthermore, there is an information gap about the molecular mechanisms of *Plasmodium falciparum*’s drug resistance, which makes it difficult to develop monitoring systems. Most countries lack adequate and comprehensive information on antimalarial drug efficacy. *Plasmodium falciparum* has developed resistance to almost all anti-malarial drugs, which poses a significant danger to malaria control worldwide. The fundamental mechanism of artemisinin resistance is due to point mutations in the beta-propeller domain of the gene encoding Kelch protein 13. Atovaquone resistance can be caused by a variety of mutations in the cytochrome b gene, with the majority of mutations affecting the protein’s ubiquinol binding site. Similarly, mutations in the *Plasmodium falciparum* chloroquine resistance transporter, *Plasmodium falciparum* multi-drug resistance 1, and an increase in *Plasmodium* falciparum Plasmepsin II and III copy numbers all lead to 4-aminoquinoline drug resistance. Also, the number of amino acid substitutions in dihydrofolate reductase and dihydropteroate synthase is correlated with the degree of antifolate drug resistance. Moreover, amino alcohol drug resistance is caused by *Plasmodium falciparum* multidrug resistance protein 1 and *Plasmodium falciparum* Na+/H + exchanger 1 mutations. In general, *Plasmodium falciparum* chloroquine resistance transporter, *Plasmodium falciparum* multidrug resistance protein 1, *Plasmodium falciparum* Na+/H + exchanger 1, plasmepsin II & III, cytochrome b gene, dihydrofolate reductase, *Plasmodium falciparum* ATPases 6, *Plasmodium falciparum* Kelch protein 13, and dihydropteroate synthase were just the molecular markers of drug resistance of *Plasmodium falciparum*. Future research on the molecular mechanisms of drug resistance in *P. falciparum* should focus on significant area including using transcriptomic and genomic technologies to identify genetic variations associated with resistance. Finding the protein interactions that underlie these resistance mechanisms requires proteomic research. Additionally, the possibility of resistance development may be decreased by investigating combination therapies that target several phases of the *P. falciparum* lifecycle. In order to successfully address drug resistance in malaria, it will be essential to strengthen worldwide monitoring systems and promote interdisciplinary collaboration among researchers and healthcare professionals. Furthermore, regular monitoring, identification, and limiting of drug-resistant *P. falciparum* strains through in vivo efficacy tests, in vitro tests, combination therapy, molecular techniques, and appropriate policies must continue to ensure the effectiveness of malaria treatment.

## Introduction

Malaria is a disease caused by a protozoan parasite of the *genus Plasmodium*. The five *Plasmodium* species that cause human malaria infection are *Plasmodium falciparum, Plasmodium vivax, Plasmodium ovale, Plasmodium malariae,* and *Plasmodium knowlesi* (Malaria 2014). Among these species, *P. falciparum* is primarily responsible for the majority of malaria-related mortalities and serious diseases. It causes more than 90% of malaria fatalities globally, making it an ongoing threat to public health on a global scale (Abebe et al. 2025; Abebe et al. 2025).

*Plasmodium falciparum* must go through a hepatocyte developmental stage. Sporozoites produced from the salivary gland of a mosquito must effectively target and penetrate hepatocytes (Frevert et al. 2005). The two main parasite ligands for liver cell invasion are the circumsporozoite protein (CSP) and the thrombospondin-related adhesion protein expressed on the sporozoite surface (Spiegel et al. 2003). Merozoites released from ruptured liver cells invade erythrocytes through binding to the surface of the erythrocyte, apical reorientation, discharge of the parasite receptor, junction reformation, and membrane penetration. These activities involve a number of merozoite-related proteins, including merozoite surface proteins (MSPs), erythrocyte-binding protein (EBA-175), and apical membrane antigen-1 (AMA-1) (Wahlgren and Perlmann 2003).

Antifolates such as pyrimethamine, proguanil, and sulfadoxine act as schizonticides in the blood. The synthesis of amino acids and nucleic acids requires the conversion of dihydrofolate to tetrahydrofolate, which is inhibited by pyrimethamine and proguanil. Sulfadoxines prevent the synthesis of dihydrofolic acid by blocking dihydropteroate synthase (Nzila 2011). *Plasmodium falciparum's* mature and early ring stages can be treated with artemisinin and its derivatives, dihydroartemisinin, artemether, and artesunate, which act against chloroquine and mefloquine-resistant strains (Okell et al. 2008). The only antimalarial drug currently recommended by the World Health Organization for use as a gametocytocidal therapy is primaquine (Dicko et al. 2018).

Artemisinin-based combination therapy and vector control interventions have significantly reduced the global burden of *P. falciparum* (Bhatt et al. 2015). However, malaria is still uncontrollable due to a number of factors, including the emergence of a drug-resistant parasite, a pesticide-resistant mosquito vector, and the lack of an efficient malaria vaccine (Thu et al. 2017).

Due to the absence of effective vaccines, management of *P. falciparum* malaria has depended largely upon chemotherapy and chemoprophylaxis (Eyasu 2015). *Plasmodium falciparum* has developed resistance mechanisms to almost all antimalarial drugs currently available, posing a significant danger to malaria control worldwide (Yobi et al. 2021). This drug resistance mechanism can be achieved in a number of ways, including alteration of drug transport and permeability, drug conversion into a form with reduced activity, increased expression of the drug's target, modifications to the drug target that reduce the binding affinity to the inhibitor, and the capacity to enter a quiescent state, in which normal cell cycle progression continues after the drug concentration is eliminated (Codd et al. 2011).

Antimalarial drug resistance in *P. falciparum* typically first appears in areas with low transmission, such as Southeast Asia or South America, before spreading to areas with high transmission, such as sub-Saharan Africa (White 2004). Antimalarial drug resistance in malaria parasites spreads because it provides a survival advantage in the presence of the antimalarial treatment, resulting in a higher likelihood of transmission for resistant parasites than for sensitive parasites. As a result, resistant infections are more likely to reoccur, and as resistance increases, infections containing resistant parasites respond more slowly to therapy. In comparison to drug-sensitive infections, increasing rates of recrudescence and slow initial treatment responses enhance the possibility of developing sufficient gametocyte densities to transmit (Menard and Dondorp 2017; Ippolito et al. 2021).

Multidrug-resistant *P. falciparum*, including resistance to structurally similar antimalarial aminoquinolines such as chloroquine, quinine, and mefloquine, is still a problem (Wongsrichanalai et al. 2002). The *P. falciparum* multidrug resistance protein 1 (PfMDR1) gene in *P. falciparum* has been linked to parasite susceptibility to a number of antimalarial drugs, including chloroquine, lumefantrine, amodiaquine, mefloquine, quinine, and artemisinin (Al-Mekhlafi et al. 2022). *Plasmodium falciparum* parasite mutations in the dihydrofolate reductase (DHFR) and dihydropteroate synthase (DHPS) genes have been linked to resistance to antifolate drugs (Sridaran et al. 2010). Resistance to quinoline-based drugs, such as chloroquine and mefloquine, is thought to depend on mutations in the *P. falciparum* chloroquine resistance transporter and PfMDR1 genes, which lead to the exclusion of the drug from the site of action (Cravo et al. 2006).

Today, the in vivo efficacy technique, the in vitro technique, and molecular methods are three different and complementary approaches used to detect antimalarial drug resistance (Nsanzabana et al. 2018). Moreover, Next generation sequencing (NGS), Sanger sequencing, real-time polymerase chain reaction (RTPCR), nucleic acid lateral flow immunoassay (NALFIA), and Q‑POC are molecular methods which are important to identify and track molecular markers of antimalarial drug resistance (Nsanzabana et al. 2018; Talundzic et al. 2018). Many factors influence the emergence of drug resistance in *Plasmodium*, including the parasite's mutation rate, the overall parasite load, the drug selection strength, and treatment compliance (Petersen et al. 2011).

Malaria control efforts on a global scale are in danger due to the emergence and spread of drug-resistant malaria. Despite stakeholders' dedication to the prevention and treatment of malaria, the current state of global health does not offer an effective answer to the issue of drug resistance (Thomas 2014). Since malaria is mostly endemic in poor countries, cost is the most profound challenge for conducting the surveillance and identification of resistant parasites (Hodoameda 2021). Furthermore, there is an information gap about the molecular mechanisms of *P. falciparum*'s drug resistance, which makes it difficult to develop monitoring systems. Most countries lack adequate and comprehensive information on antimalarial drug efficacy. This causes chemoprophylaxis and treatment of malaria to be more compromised in those resource-poor settings, resulting in suboptimal antimalarial treatment policies (Plowe 2003).

It is urgently necessary to have a better knowledge of the molecular mechanisms underlying drug resistance in order to reduce or stop the propagation of resistance, to more effectively use local treatments, to extend the shelf life of existing drugs, and to create new medications (Hayton and Su 2008). Moreover, studying molecular markers of antimalarial drug resistance is used to detect the emergence of resistance and assess its spread (Organization 2010). It also gives information on the genetics of parasites linked to resistance, such as single nucleotide polymorphisms or differences in gene copy number that lead to a reduction in parasite susceptibility to antimalarial drugs. Therefore, tracking the emergence and/or spread of antimalarial drug resistance is made possible by the detection of molecular markers (Organization 2015a). Therefore, the aim of this review was to assess molecular mechanisms of drug resistance and markers of *P. falciparum.*

## Molecular mechanisms of drug resistance of *Plasmodium falciparum*

### Artemisinin drug resistance mechanisms

Artemisinin is a potent and rapidly acting blood schizontocide and is active against all *Plasmodium* species (Organization 2015a). It causes a very rapid decrease in parasitemia that begins very immediately after treatment and kills all stages of the malaria parasite, including immature gametocytes (Akompong et al. 2000). The cleavage of the endoperoxide bridge in artemisinin by free Fe (II) protoporphyrin IX, which is released from digested hemoglobin, is the mechanism by which it kills parasites. The heme-carbon-centered drug's radical alkylates heme, proteins, and lipids once it has been activated, speeding up the production of more cytotoxic reactive oxygen species through a cluster bomb effect that eventually causes the parasite to die (Ismail et al. 2016).

For artemisinin resistance to be confirmed, four criteria must be met. These include parasite reemergence within 28 days of starting treatment, sufficient plasma concentrations of dihydroartemisinin, a longer parasite clearance time, and reduced parasite susceptibility (Noedl 2005). The first stage of artemisinin resistance is a delay in parasite clearance time after therapy with artemisinin combination therapies. According to the World Health Organization, artemisinin resistance is characterized by an increase in parasite clearance time, treatment failure after treatment with an oral artemisinin-based monotherapy with acceptable antimalarial blood concentration, and recrudescence within 28–42 days (Organization 2010).

Artemisinin susceptibility is reduced solely in ring-stage parasites, which is a distinctive characteristic of artemisinin-resistant parasites (Witkowski et al. 2010). Artemisinin resistance has been associated with numerous molecular mechanisms and targets (Krishna et al. 2014). The basic mechanism of artemisinin resistance is due to point mutations in the beta-propeller domain of the gene encoding Kelch protein 13 (*Pf*k13), which are probably responsible for reduced susceptibility to artemisinin and its derivatives (Ouji 2018). Reduced artemisinin interactions with *P. falciparum* phosphatidylinositol-3-kinase (*Pf*PI3K) are the result of mutant Kelch 13 (Bhattacharjee et al. 2012).

Artemisinin resistance is more prevalent in many countries, including Cambodia, Vietnam, Myanmar, Thailand, and Laos (Menard and Dondorp 2017). Artemisinin combination treatment (Patel et al. 2012) has taken the place of quinolines and antifolates as the first-line therapy for uncomplicated *P. falciparum* malaria.

Artemether/lumefantrine(AL),aresunate/amodiaquine(ASAQ),artesunate/mefloquine,artesunate/sulfadoxine/pyrimethamine, and dihydroartemisinin/piperquinine are the five ACTs currently in use (April 2017). Additionally, with ACT therapies, a number of factors, including initial parasite biomass, partner drug efficacy, patient age, health status, and artemisinin dose, could affect parasite clearance time values (Simwela et al. 2020). The appearance and/or spread of malaria parasites resistant to ACT in areas of the world with high malaria burdens, such as sub-Sahara Africa countries and India, is of great concern (Chookajorn 2018). With regard to treatment failure after the ACT-artesunate + sulfadoxine/pyrimethamine combination, high frequencies of PfDHFR and PfDHPS mutations have been reported (Abdifatah et al. 2018).

In the case of artemisinin resistance, four new hypotheses have been proposed. These are increased production of phosphatidylinositol-3-phosphate (PI3P), upregulation of the ubiquitin/proteasome system, activation of the unfolded protein response pathway, and a reduction in the production of damaged proteins (Noedl 2005).

#### Increase in phosphatidylinositol-3-phosphate production

*Plasmodium falciparum* phosphatidylinositol 3-kinase*,* which phosphorylates *P. falciparum *phosphatidylinositol to generate PI3P, is inhibited by dihydroartemisinin (Mbengue et al. 2015). Cell signaling and survival in organisms are mediated by PI3P (Davis et al. 2015). The decrease in PI3P concentrations caused by artemisinin treatment in artemisinin-susceptible *P. falciparum* parasites leads to insufficient activation of cell survival signaling pathways. However, the concentration of PI3P in artemisinin-resistant parasites is higher than in artemisinin-susceptible parasites, implying that PI3P-mediated signaling is involved in artemisinin resistance (Mbengue et al. 2015).

#### Upregulation of ubiquitin/proteasome system

Protein translation is inhibited by oxidative stress, and protein breakdown is induced by the ubiquitin/proteasome system. The ubiquitin/proteasome system is up-regulated in artemisinin-resistant parasites compared to artemisinin-susceptible parasites (Mok et al. 2011; Dogovski et al. 2015). Protein ubiquitination is higher in trophozoite-infected red blood cells following dihydroartemisinin treatment than in uninfected red blood cells, suggesting that protein ubiquitination is driven by dihydroartemisinin-induced cellular stress. In the artemisinin-resistant parasites, however, upregulation of the ubiquitin/proteasome system is likely to result in rapid degradation of the damaged proteins (Dogovski et al. 2015).

#### Activation of unfolded protein response pathway

Transcriptomics profiling is used to investigate the essential aspects of artemisinin resistance by comparing gene expression between resistant lines produced in vitro with and without continuous artemisinin pressure (Witkowski et al. 2010). Exported proteins, such as the *P. falciparum* knob-associated histidine-rich protein and repetitive interspersed family proteins, as well as chaperones and cell cycle regulators, were the most up-regulated genes in resistant parasites. In erythrocytic asexual stages, artemisinin-resistant parasites showed massive changes in the transcriptional cascade (Mok et al. 2011).

When comparing artemisinin-resistant parasites to artemisinin-susceptible parasites, gene expression associated with basic metabolic and cellular pathways in the ring stage is greatly delayed in artemisinin-resistant parasites (Dogovski et al. 2015). The unfolded protein response is known to be a coping mechanism in response to endoplasmic reticulum stress. It is heavily involved in the functional pathways involving the identified up-regulated genes. This is implies that artemisinin resistance mitigates the potential damage of parasite proteins by artemisinin (Hetz et al. 2015; Hetz Flores et al. 2015).

#### Lower production of damaged proteins

**Fig. 1:**
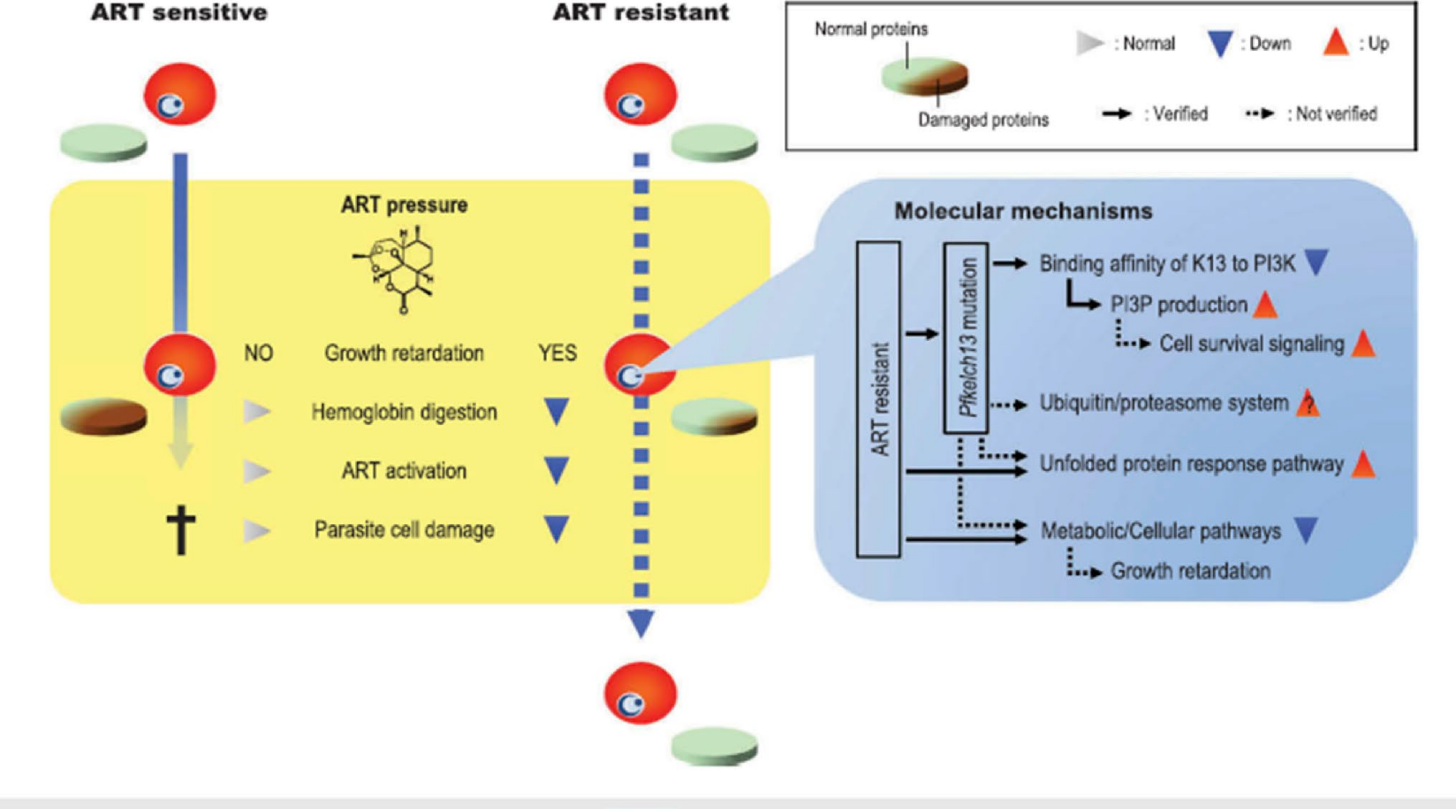
Mechanisms of artemisinin resistance in *Plasmodium falciparum*

Damaged proteins may accumulate beyond the parasite's ability to maintain proteostasis in artemisinin-resistant parasites (Mita et al. 2016). This carryover of damaged proteins has the potential to kill parasites. Artemisinin resistance slows parasite growth in the ring stage. Therefore, artemisinin may be less activated due to reduced hemoglobin digestion than in artemisinin susceptible parasites. As a result, the amount of damaged proteins could be kept within a sustainable level of proteostasis, perhaps allowing parasites to survive (Fig. 1) (Dogovski et al. 2015).

### Atovaquone drug resistance mechanisms

Atovaquone has been used to treat malaria since the 1980s. Atovaquone, a substituted hydroxynaphthoquinone, is an effective antimalarial drug that acts by blocking the parasite's mitochondrial cytochrome bc1 complex from functioning (Birth et al. 2014). As a malaria prophylaxis or treatment, atovaquone is combined with proguanil (Nakato et al. 2007).

Atovaquone resistance can be caused by a variety of mutations in the cytochrome b gene, with the majority of mutations affecting the protein's ubiquinol binding site (Gil et al. 2003; Korsinczky et al. 2000). Resistance to atovaquone has been linked to cytochrome b Tyr 268Ser/Cysteine/Asn mutations (Mather et al. 2005). When combined with another drug, such as proguanil or tetracycline, resistance to atovaquone develops more slowly than when taken alone (Fisher et al. 2012).

### 4-Aminoquinolines drug resistance mechanisms

#### Chloroquine drug resistance mechanisms

Malaria caused by *P. falciparum* with chloroquine resistance first appeared in Thailand in 1957. After spreading through South and Southeast Asia, they appeared in sub-Saharan Africa and South America by the 1970s. Notably in Sub-Saharan Africa, the increase in chloroquine resistance caused a global increase in malaria-related mortality (Packard 2014). The blood stage of *P. falciparum* ingests hemoglobin from the host erythrocyte as a major source of amino acids. Chloroquine prevents the parasite from developing by blocking the detoxification of heme, a toxic by-product produced by the parasite's digestion of hemoglobin in the parasite's food vacuole. The amount of chloroquine in the food vacuole is linked to the drug's efficacy (Mita and Tanabe 2012).

Chloroquine levels are higher in susceptible parasites than what they are in resistant parasites. The key determinant for chloroquine resistance in *P. falciparum* is mutations in the PfCRT gene, which encodes a 49-kilodalton (kDa) protein (Golassa et al. 2015) with 424 amino acids (Fidock, et al. 2000). The chloroquine resistant transporter of *P. falciparum* is found in the parasite's food vacuole and has ten putative transmembrane domains (Cooper et al. 2007). *Plasmodium falciparum* chloroquine-resistant transporter maintains osmotic balance across the food vacuole membrane, possibly via transporting hemoglobin digestion products and ions (Martin, et al. 2009; Roepe 2011). Chloroquine resistance is produced by PfCRT through a variety of processes, including the use of energy to move CQ out of the drug DV (Sanchez et al. 2005), the facilitation of charged drug species diffusion (Bray et al. 2005), and pH changes to the DV (Bennett et al. 2004).

**Fig. 2:**
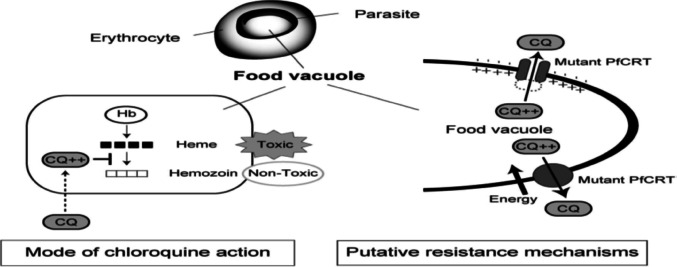
Putative mechanism of *Plasmodium falciparum* chloroquine resistance

Chloroquine resistance is conferred by a lysine (Lys) to threonine (Thr) amino acid substitution at codon 76 Lys76Thr of PfCRT, and the altered protein results in enhanced efflux of chloroquine from the food vacuole and a reduction in chloroquine concentration (Sanchez et al. 2003). *Plasmodium falciparum* chloroquine resistance transporter (Golassa, et al. 2015) 76 T point mutation is the primary driver of chloroquine resistance (Dajem et al. 2012). And also, mutations from asparagines (Asn) to tyrosine (Tyr) in codon 86 of the multidrug-resistant gene and copy number changes in another gene (Pfmdr1) that encodes a homolog of the human multi-drug resistance p-glycoprotein (PfPgh1) were associated with chloroquine resistance (Hayton and Su 2008). The amino acid substitution N86Y (PfMDR1), common in African strains, modulates drug susceptibility by enhancing parasite resistance to chloroquine (Veiga et al. 2016). Mutations of PfCRT and PfMDR1 were associated with chloroquine resistance (Fig. 2) (Hussien et al. 2020).

#### Amodiaquine and piperaquine resistance mechanisms

Amodiaquine has structural similarities with chloroquine and has a short half-life of 3 h; therefore, it is believed that the antimalarial activity is mediated by the primary metabolite, monodesethylamodiaquine, which has a half-life of 9–18 days (Stepniewska and White 2008; Sá et al. 2009). Amodiaquine has been shown to accumulate in the digestive vacuole (L’Episcopia et al. 2021), bind to heme, and prevent the process of detoxifying heme (Hayeshi et al. 2008). Resistance to amodiaquine is caused by mutations in PfCRT and PfMDR1 (Sá et al. 2009). The amino acid substitution N86Y (PfMDR1), common in African strains, modulates drug susceptibility by enhancing parasite resistance to amodiaquine (Veiga et al. 2016).

Piperaquine is a bis-4-aminoquinoline with a half-life of about 5 weeks. Piperaquine has been hypothesized to have a similar mode of action to chloroquine due to structural similarities. Piperaquine accumulates in the DV and is a strong inhibitor of heme polymerization, despite the fact that the specific mechanism of action is unknown (Warhurst et al. 2007). In China, *P. falciparum* that is piperaquine resistant has been reported (Davis et al. 2005). And also, piperaquine resistance has been linked to an increase in *P. falciparum* Plasmepsin II and III (Pfmp2 and Pfmp3) copy numbers (Bopp et al. 2018). In addition, mutations in the PfCRT gene cause piperaquine susceptibility to be modulated (Petersen et al. 2011). Genome-wide association studies and clinical trials have associated increased copy number of the genes encoding the hemoglobinases plasmepsin II and plasmepsin III with piperaquine resistance (Loesbanluechai et al. 2019).

### Antifolate drug resistance mechanisms

Early on, after their introduction in the 1970s, resistance to antifolates was first reported in South America and Southeast Asia. Now, in both locations, the parasite population has developed high-level antifolate resistance due to mutations (Heinberg and Kirkman 2015). Antifolates like pyrimethamine and sulfadoxine inhibit the growth of *P. falciparum* by blocking the pathway where folate is produced (Hyde 2005).

Dihydrofolate reductase, an enzyme that is encoded by the DHFR gene, reduces dihydrofolate to tetrahydrofolate, a cofactor that is necessary for the production of methionine and nucleic acids (Askari and B. and M. Krajinovic 2010). Pyrimethamine resistance has been associated with DHFR mutations at positions 50, 51, 59, 108, and 164. A serine (Ser)-to-asn substitution at position 108 is the first step toward increased resistance. Overall, the number of substitutions in DHFR causes a rise in the level of pyrimethamine resistance (Sharma and Chauhan 2012).

The dihydropteroate synthase gene encodes sulfadoxine, a potent inhibitor of the dihydropteroate synthase enzyme. Mutations at positions 436, 437, 540, 581, and 613 decrease the affinity of dihydropteroate synthase for sulfadoxine. The degree of sulfadoxine resistance is frequently correlated with the number of amino acid substitutions in DHPS (Mita and Tanabe 2012). An alanine (Ala) to glycine (Gly) substitution at position 437 has been proposed as the first step toward sulfadoxine resistance (Basuki et al. 2014).

Treatment with pyrimethamine or sulfadoxine commonly causes a rise in gametocytosis. Increased gametocytogenesis may have contributed to greater malaria transmission, which is probably related to the rapid spread of pyrimethamine or sulfadoxine resistance (Barnes et al. 2008; Stepniewska et al. 2008). According to a report, PfDHFR and PfDHPS mutant alleles with pyrimethamine and sulfadoxine resistance are common (Tuedom, et al. 2021).

### Amino alcohol drug resistance mechanisms

#### Lumefantrine and mefloquine resistance mechanisms

Lumefantrine is structurally similar to antimalarials that are hydrophobic arylamino alcohols. It has a half-life of 3–5 days, and absorption of this lipophilic drug varies from person to person (Schlitzer 2007). Amplification of the encoding gene, (PfMDR1) and polymorphisms in PfMDR1, particularly the variation Asn86, have been linked to lower susceptibility to lumefantrine (Conrad and Rosenthal 2019).

Mefloquine is a 4-methanolquinoline with a 14–18 day half-life (Stepniewska and White 2008). Midway through the 1980s, mefloquine was first made available in Thailand as a first-line antimalarial, but resistance soon developed (Wongsrichanalai et al. 2002). Mefloquine can bind to heme, as shown by in vitro tests, and exert some antimalarial activity by preventing heme detoxification (Eastman and Fidock 2009). Amplification of Pfmdr1, resulting in overexpression of this resident DV membrane transporter, mediates mefloquine resistance (Petersen et al. 2011). *Plasmodium falciparum* multidrug resistance-1 Ser1034, Asn1042, and Aspartate (Asp) 1246 mutations also cause mefloquine resistance (Purfield 2007). Studies conducted in vitro show that Pfmdr1 gene amplification is a key factor in mefloquine resistance and is linked to a higher probability of treatment failure (Alker et al. 2007).

#### Quinine drug resistance mechanism

Quinine is one of the oldest antimalarial agents and is still effective in treating *P. falciparum* (Petersen et al. 2011). Although its efficacy is declining in some endemic regions (Pukrittayakamee et al. 2000). Quinine is now used to treat severe cases of malaria and as a second-line treatment for resistant malaria when combined with antibiotics. Because of its short half-life of 8–10 h, widespread quinine resistance is rare (Pascual et al. 2013). The molecular mechanism by which quinine acts against *P. falciparum* is by accumulating in the parasite’s DV and preventing the detoxification of heme. Although numerous molecules have been shown to play a role in parasite responses to quinine, the molecular basis of quinine drug resistance (QNR), as defined by an increase in the in vitro inhibitory concentration 50 (IC50), remains unknown (Fitch 2004). Higher levels of IC50 in children of a genetic cross have been linked to the PfCRT, a gene encoding a putative *P. falciparum* sodium/hydrogen exchanger (PfNHE), Pfmdr1, and a location on chromosome nine (Ferdig et al. 2004).

The involvement of PfNHE in QNR is further supported by enhanced PfNHE activities in parasites with high levels of quinine IC50. The role of Pfmdr1 in QNR is consistent with the report that the Asn1042Asp substitution in PfPGH1 contributed to QNR (Roepe et al. 2009). Both PfPGH1 and PfNHE may regulate cytosolic and/or vacuolar pH, resulting in drug accumulation changes. Other unknown transporters, particularly adenosine triphosphate (Pluijm et al. 2020)-binding cassette (ABC) transporters, also may contribute to QNR. Because the *P. falciparum* parasite response to quinine is likely a multi-gene characteristic, the necessity of many loci for QNR may explain why quinine is still effective in treating malaria parasites (Hayton and Su 2008).

## *Plasmodium falciparum *molecular markers of drug resistance

### Molecular markers of resistance to quinoline-based drugs

#### *Plasmodium falciparum* multidrug resistance protein 1

*Plasmodium falciparum* multidrug resistance protein 1 belongs to the ABC. P-glycoprotein homolog 1 is another name for Pfmdr1 (PGH-1) (Hodoameda 2021). This gene, which is also found on *P. falciparum's* chromosome 5, generates a 160-kDa P-glycoprotein that aids in drug efflux (Ferdig et al. 2004). *Plasmodium falciparum* multidrug resistance protein 1 is a transporter that regulates drug accumulation in the parasite's DV (Hodoameda 2021). Under the influence of chloroquine, the expression of the PfMDR1 gene is regulated differently, with a higher amount of PfMDR1 transcription (Myrick et al. 2003). *Plasmodium falciparum* multidrug resistance protein 1 is involved in the parasite's response to various antimalarial drugs. Increased PfMDR1 copy number or mutations in the gene are two mechanisms used by the Pfmdr1 gene to regulate antimalarial drug responsiveness (Duah et al. 2013).

Chloroquine resistance has been linked to mutations in the Pfmdr1 gene at codons 86, 184, 1034, 1042, and 1246 (ElBadry et al. 2013). The Asn to Tyr mutation at codon 86 has been widely utilized among these mutations (Fidock et al. 2000). Mefloquine monotherapy is caused by a rise in the number of copies of Pfmdr1 in clinical isolates (Nelson et al. 2005) or artesunate-mefloquine combination treatment failures (Alker et al. 2007). Resistance to antimalarial drugs is caused by polymorphisms seen in various haplotypes of PfMDR1. The substrate specificity of PfMDR1 is altered by these mutations (Ferreira et al. 2011). The Asn86 Tyr mutation in Pfmdr1 has been linked to chloroquine and amodiaquine treatment failure; however, the link to chloroquine is weak (Picot et al. 2009).

Resistance to lumefantrine is linked to the Pfmdr1 Asn86-Phenylalanine184-Asp1246 haplotype (Nsobya et al. 2006). The PfMDR1 Ser1034Cysteine/Asn1042Asp/Asp1246Tyr mutations are linked with reduced susceptibility to quinine (Reed et al. 2000). The PfMDR1 and PfCRT alleles may interact to increase amodiaquine and desethyl-amodiaquine (DEAQ) resistance (Sá et al. 2009). Although the multidrug resistance protein 1 of *P. falciparum* alone is insufficient to provide chloroquine resistance, it may aid in the development of drug resistance (Reed et al. 2000).

#### *Plasmodium falciparum* chloroquine resistance transporter

The *Plasmodium falciparum* chloroquine resistance transporter gene is a putative transporter that weighs 49 kDa, belongs to the drug transporter superfamily, and is found in the parasite DV (Fidock, et al. 2000; Martin and Kirk 2004). This gene, which encodes a protein called PfCRT, was also found on chromosome 7. Chloroquine resistance is mostly caused by mutations in the Pfcrt gene (Fidock et al. 2000; Ahn et al. 2006). For the purpose of assessing the structural alterations of the PfCRT gene, three parameters have been taken into account: aggregation prediction, amyloid prediction, and chaperone-binding prediction (Baets et al. 2012).

Chloroquine-resistant parasites have a mutation at codon 76 (Lys to Thr) (Fidock et al. 2000). In addition to the Lys76Thr mutation in Pfcrt, mutations in codons 72, 74, 75, 97, 220, 271, 326, 356 and 371 have been linked to chloroquine resistance (Fidock et al. 2000). Individuals' drug absorption and metabolic rate will also have an impact on the outcome of chloroquine treatment (Sidhu et al. 2002; Cooper et al. 2002).

#### *Plasmodium falciparum* multidrug resistance-associated protein

The multidrug resistance-associated protein of *P. falciparum* (Pfmrp) is a member of the ABC transporter family. *Plasmodium falciparum* multidrug resistance-associated protein is a transport regulator. Resistance to antimalarial drugs such as quinine and chloroquine has been linked to mutations in the *P. falciparum* multidrug resistance-associated protein (Mu et al. 2003).

#### *Plasmodium falciparum* Na + /H + exchanger 1

The *Plasmodium falciparum* sodium-hydrogen exchanger 1 gene of *P. falciparum is* a putative Na + /H + exchanger located on chromosome 13 of the parasite genome. Additionally, as a response to acidification by anaerobic glycolysis, the parasite's primary source of energy, PfNHE-1 is involved in active proton efflux to maintain a PH of 7.4 within the parasite (NDUNG’U, 2018). Some polymorphisms in the PfNHE-1 gene are linked to antimalarial drug resistance, like quinine, whereas others lead to enhanced susceptibility to other antimalarial drugs (Ferdig et al. 2004). The PfNHE-1 of *P. falciparum* contributes to quinine resistance in parasites in a strain-specific manner, and other parasite genetic background factors are also required for quinine resistance in parasites (Ekland and Fidock 2007).

#### Plasmepsin II &III

*Plasmodium falciparum* plasmepsins are aspartic proteases involved in the breakdown of hemoglobin. They have a weight of about 38 kDa. In the parasite's DV, Pfmp2 and Pfmp3 cleave hemoglobin (Banerjee et al. 2002). Piperaquine, an aminoquinoline drug, targets Pfmp2 and Pfmp3 to inhibit them as its mode of action. Piperaquine resistance has been linked to an increase in Pfmp2 and Pfmp3 copy numbers (Bopp et al. 2018). The plasmepsin II and III genes, which are found on chromosome 14 of *P. falciparum*, may function as a molecular marker for reduced piperaquine sensitivity, according to two recent studies (Witkowski et al. 2017; Amato et al. 2017).

### Molecular markers for resistance to antifolates

#### Dihydrofolate reductase

Pyrimethamine blocks *P. falciparum's* DHFR enzyme and consequently its folate biosynthesis pathway. Under the influence of pyrimethamine, parasites can upregulate the translation but not the transcription of DHFR to counteract its effect (Nirmalan et al. 2004). *P. falciparum* DHFR has a crystal structure, and the pyrimethamine binding sites have been identified (Yuvaniyama et al. 2003; Sirawaraporn et al. 2002). However, mutations in some of the enzyme's key amino acids cause it to have a lower affinity for the drug (Bras and Durand 2003).

It's widely known that changing serine to ASN at codon 108 of the *P. falciparum* DHFR decreases drug susceptibility. This mutation showed pyrimethamine resistance. The isoleucine-164-leucine (I164L) mutation is found to play a significant role among these mutations, as its association with other DHFR mutations always resulted in a higher level of drug resistance (Ménard et al. 2008).

#### Dihydropteroate synthase

Sulphadoxine functions as a competitive inhibitor in the parasite's folate biosynthesis pathway by mimicking p-aminobenzoic acid. This drug works by interfering with the conversion of dihydropteridine pyrophosphate to dihydropteroate by inhibiting the enzyme DHPS (Franklin and Snow 2005).

However, due to mutations in the parasite enzyme DHPS, the parasite has developed resistance to sulphadoxine (Heinberg and Kirkman 2015). Several key point mutations in this parasite enzyme have been found, which can decrease its drug binding affinity (Heinberg and Kirkman 2015). Codons 436 Ser to Ala/Phenylalanine (Ser to Ala/Phe), 437 Ala to Gly, 540 Lys to Glutamate (Lys to Glu), 581 Ala to Gly, and 613 Ala to Ser/Thr have the most changes. The increasing degree of sulphadoxine drug resistance has been linked to a higher number of mutations in DHPS, similar to DHFR. The Ala437Gly mutation in DHPS is a crucial point mutation that permits the parasite to decrease its sulfadoxine susceptibility (Ahmed et al. 2004).

### Molecular markers of resistance to artemisinin

In most malaria-endemic countries, artemisinin and its derivatives are the most commonly used antimalarial drugs. The emergence of full resistance to artemisinin and its derivatives is the result of delayed parasite clearance (Organization 2015b).

The ATP-consuming calcium (Ca)-dependent *P. falciparum* sarco/endoplasmic reticulum Ca2 + -ATPases 6 has been suggested as a molecular marker for partial resistance to artemisinin and its derivatives. The Kelch 13 gene is another gene that has been linked to artemisinin and its derivatives. The Kelch 13 gene encodes 726 amino acids and is found on chromosome 13. The kelch protein family has many activities, including protein organization and interaction with other proteins (Ariey et al. 2014).

### Molecular markers of resistance to atovaquone

Atovaquone interacts with the cytochrome b1 complex to prevent electron transport in the mitochondria (Hughes et al. 2010). As a result, it is possible to use the cytochrome b gene as a molecular marker to monitor atovaquone resistance (Wilkinson et al. 2003). The cytochrome bc1 complex catalyzes the transfer of electrons from ubiquinol to cytochrome c, which is related to the translocation of protons across the inner mitochondrial membrane. This ensures that the membrane potential of the mitochondria is used by an ATP synthase to produce ATP. By causing a collapse in the mitochondrial membrane potential, the antimalarial drug kills the parasite (Barton et al. 2010).

Mutations in the cytochrome b gene can lead to atovaquone resistance, with most of these mutations altering the protein's ubiquinol binding region (Gil et al. 2003; Korsinczky et al. 2000). The mutations in the ubiquinol binding sites have been hypothesized to confer a fitness cost to the parasite in the absence of atovaquone drug pressure (Table 1) (Barton et al. 2010).

**Table 1:** Summary of antimalarial drugs and their molecular markers of resistance

Antimalarial drug	Molecular markers of resistance
Quinine	PfMDR1, Pfmrp, and PfNHE-1
Mefloquine	PfMDR1
Lumefantrine	PfMDR1
Chloroquine	PfCRT, PfMDR1, and Pfmrp
Amodiaquine	PfCRT and PfMDR1
Piperaquine	Pfpm2 and Pfpm3
Pyrimethamine	PfDHFR
Sulfadoxine	PfDHPS
Artemisinin	Pfk13 and Pfatp6
Atovaquone	Pfcytb

## Conclusion

The extensive study of the molecular mechanisms of drug resistance of the malaria parasite, as well as the use of molecular markers of resistance to monitor the emergence and spread of parasite resistance to antimalarial medications, is a highly effective method of monitoring drug resistance. Increased production of PI3P, upregulation of the ubiquitin/proteasome system, activation of the unfolded protein response pathway, and a reduction in the production of damaged proteins are the four new hypotheses of artemisinin resistance. Resistance to the endoperoxide artemisinins is mediated by mutations in K13. Atovaquone resistance can be caused by a variety of mutations in the cytochrome b gene. Mutations in the transmembrane proteins PfCRT and PfMDR1 are primarily responsible for the mechanisms of resistance to 4-aminoquinolines and amino alcohol drugs that target parasite DV processes such as hemoglobin breakdown and hemozoin production. Pyrimethamine and sulfadoxine resistance have been associated with DHFR and DHPS mutations, respectively. In general, several molecular markers of drug resistance of *P. falciparum* are included, like PfCRT, PfMDR1, Pfmrp, Pfatp6, Pfk13, PfNHE1, the cytochrome bc1 complex, DHFR, and DHPS.

Studies on the molecular mechanisms of drug resistance in *P. falciparum* should focus on certain key areas in the future. The identification of certain genetic variants linked to resistance to antimalarial medications will be made possible by the use of genomic and transcriptomic technologies. Furthermore, identifying the protein interactions that contribute to resistance mechanisms requires the use of proteomic investigations.

Additionally, investigating combination treatments that focus on different stages of the *P. falciparum* lifecycle may lower the likelihood of resistance development. Finding trustworthy biomarkers for early resistance diagnosis is essential for making therapy modifications on time. Improving international surveillance systems will make it easier to track drug-resistant strains and enable prompt public health measures. Lastly, encouraging multidisciplinary cooperation between researchers and medical professionals would guarantee a thorough strategy for combating drug resistance in malaria. The scientific community may make substantial progress in understanding and managing medication resistance in malaria by exploring these areas.

Due to the widespread and quick emergence of drug resistance, different measures must be taken to prevent drug resistance with the remaining potent compounds as well as any new compounds that may be developed in the future. In addition, regular monitoring, identification, and limiting of drug-resistant *P. falciparum* strains through in vivo efficacy tests, in vitro tests, combination therapy, molecular techniques, and appropriate policies must continue to ensure the effectiveness of malaria treatment.

## Abbreviations

ABC: ATP-binding cassette
ALA: Alanine
ACT: Artemisinin combination treatment
ASP: Aspartate
ASN: Asparagines
ATP: Adenosine triphosphate
DHFR: Dihydrofolate reductase
DHPS: Dihydropteroate synthetase
DV: Digestive vacuole
GLY: Glycine
IC50: 50 Inhibitory concentration
KDA: Kilodalton
LYS: Lysine
PFCRT: *Plasmodium falciparum* chloroquine resistant transporter
PFPGH1: *Plasmodium falciparum* homolog P-glycoprotein
PFMDR1: *Plasmodium falciparum* multi-drug resistance 1
PF MP2 and MP3: *P. falciparum* Plasmepsin 2 &3
PfNHE: *Plasmodium falciparum* sodium/hydrogen exchanger
PI3P: Phosphatidylinositol-3-phosphate
QNR: Quinine drug resistance
SER: Serine
THR: Threonine
TYR: Tyrosine
